# A Cross-Sectional Investigation of Preadolescent Cardiometabolic Health: Associations with Fitness, Physical Activity, Sedentary Behavior, Nutrition, and Sleep

**DOI:** 10.3390/children10020336

**Published:** 2023-02-09

**Authors:** Nicholas Castro, Gabriel Zieff, Lauren C. Bates, Patricia Pagan Lassalle, Simon Higgins, James Faulkner, Sally Lark, Paula Skidmore, Michael J. Hamlin, T. Leigh Signal, Michelle A. Williams, Lee Stoner

**Affiliations:** 1 School of Health and Applied Human Sciences, University of North Carolina Wilmington, Wilmington, NC 28403, USA; 2 Department of Exercise and Sport Science, University of North Carolina at Chapel Hill, Chapel Hill, NC 27513, USA; 3 School of Sport, Health, and Community, University of Winchester, Winchester SO224NR, UK; 4 School of Sport, Exercise and Nutrition, Massey University, Wellington 4442, New Zealand; 5 Department of Medicine, University of Otago, Dunedin 9016, New Zealand; 6 Department of Tourism, Sport and Society, Lincoln University, Christchurch 7647, New Zealand; 7 School of Health Sciences, Sleep-Wake Research Centre, Massey University, Wellington 4442, New Zealand; 8 Department of Epidemiology, Harvard T.H. Chan School of Public Health, Boston, MA 02115, USA

**Keywords:** cardiovascular disease, metabolic disease, cardiometabolic disease, childhood, lifestyle factors

## Abstract

Background: Cardiometabolic disease (CMD) risk often begins early in life. Healthy lifestyle behaviors can mitigate risk, but the optimal combination of behaviors has not been determined. This cross-sectional study simultaneously examined the associations between lifestyle factors (fitness, activity behaviors, and dietary patterns) and CMD risk in preadolescent children. Methods: 1480 New Zealand children aged 8–10 years were recruited. Participants included 316 preadolescents (50% female, age: 9.5 ± 1.1 years, BMI: 17.9 ± 3.3 kg/m^2^). Fitness (cardiorespiratory fitness [CRF], muscular fitness), activity behaviors (physical activity, sedentary, sleep), and dietary patterns were measured. Factor analysis was used to derive a CMD risk score from 13 variables (adiposity, peripheral and central hemodynamics, glycemic control, and blood lipids). Results: Only CRF (β = −0.45, *p* < 0.001) and sedentary time (β = 0.12, *p* = 0.019) were associated with the CMD risk score in the adjusted multivariable analysis. CRF was found to be nonlinear (VO_2_ max ≤ ≈42 mL/kg/min associated with higher CMD risk score), and thus a CRF polynomial term was added, which was also associated (β = 0.19, *p* < 0.001) with the CMD risk score. Significant associations were not found with sleep or dietary variables. Conclusion: The findings indicate that increasing CRF and decreasing sedentary behavior may be important public health targets in preadolescent children.

## 1. Introduction

The prevalence of chronic cardiometabolic diseases (CMD) in children is a major public health concern. For example, according to the World Health Organization, in 2016, over 340 million children and adolescents aged 5–19 years were overweight or obese [1]. Further, a recent study of Caucasian children aged 10–12 years old reported that over 10% of children with obesity had been diagnosed with metabolic syndrome, a clustering of cardiovascular (e.g., high blood pressure) and metabolic (abdominal adiposity, high fasting blood glucose, low high-density lipoprotein cholesterol [HDL-C], high triglycerides [TG]) risk factors [2]. The early incidence of CMD risk has been associated with unhealthy yet modifiable lifestyle behaviors, including physical inactivity, sedentary behavior, unhealthy diet, and inadequate sleep [3,4,5,6]. Additionally, activity (or movement) behaviors such as physical activity and sedentary behavior are directly linked to cardiorespiratory fitness (CRF) and muscular fitness, which are also important predictors of CMD risk in preadolescents [3]. However, it is unclear whether certain lifestyle factors are more or less important than others to CMD risk in preadolescents. In order to mitigate early CMD risk, a greater understanding of how lifestyle factors impact CMD risk in preadolescents is needed.

Though preadolescence is a key developmental period, few studies have used data-driven approaches to comprehensively assess associations between lifestyle factors and CMD risk [3,4,5]. Moreover, it is particularly important to examine multiple lifestyle factors as these behaviors tend not to occur in isolation but rather cluster together and interact with one another [7,8,9,10]. For example, in the adult population, exercise engagement to increase physical activity has been shown to improve sleep quality and duration [11], and in adolescents, sitting time has been inversely related to daily fruit and vegetable consumption [12]. While our data are not sufficient to explore these interactions, the inclusion of multiple lifestyle factors in the same model is important to gain insight into these behaviors’ relative contributions to CMD risk in preadolescents.

Appropriate modification of lifestyle factors can reduce CMD risk [13,14], including in adolescents [15] and preadolescents [16]. The purpose of this cross-sectional study was to simultaneously examine which of the following behaviors is most strongly associated with CMD risk in preadolescent children: cardiorespiratory fitness (CRF), muscular fitness, physical activity, sedentary behavior, nutrition, or sleep.

## 2. Materials and Methods

This observational study was carried out in accordance with Strengthening the Reporting of Observational Studies in Epidemiology (STROBE) guidelines [17]. The methodology was prospectively outlined in Castro et al. [18]. The trial was registered with the Australian and New Zealand Clinical Trials Registry (ACTRN12614000433606).

### 2.1. Recruitment and Participants

**Figure 1:**
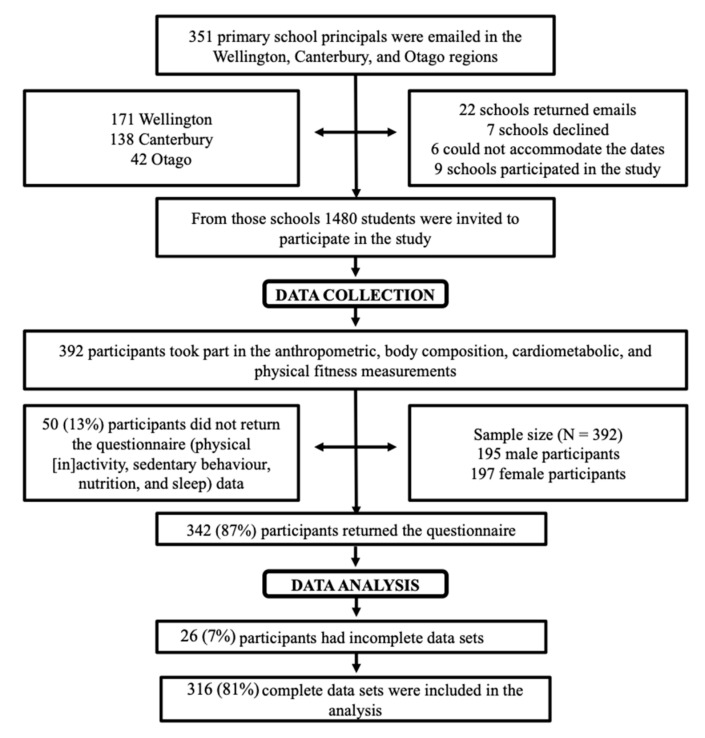
Flow Chart Showing Participant Recruitment and Study Timeline. Includes information regarding the time and location of recruitment, data collection, and analysis.

Children 8 to 10 years of age were recruited from public schools in three major cities in New Zealand between April 2015 and April 2016 (Figure 1). Stratified sampling was implemented such that within each sample location (city), regional schools were stratified by socioeconomic status, and schools from each stratum were randomly invited to participate. New Zealand public-funded schools were classified on a 10-point decile rating (until 2017) based on the major socioeconomic status of the students. Ratings ranged from low (1 to 5) to high (6 to 10) (Table 1). Decile 1 schools consisted of the 10% of schools with the greatest percentage of students from low socioeconomic status communities. Decile 10 schools consisted of the 10% of schools with the smallest percentage of students from low socioeconomic status communities.

All children were eligible to participate at the invited schools unless they had a musculoskeletal impairment or surgical procedure that did not allow full function within the previous month or were currently taking cardiovascular medicine. Based on the guidelines of the New Zealand Health and Disability Ethics Committee (HDEC), parent/guardian consent and child assent were acquired before participation. 

### 2.2. Study Design

Data collection at each school commenced on Monday and finished on the following Friday, with each testing session lasting approximately 30–45 min per participant. CMD risk was measured between 09:00 a.m. and 12:00 p.m., with participants (a) having abstained from exercise for the 24 h prior, (b) having been at least three hours fasted, and (c) being in a hydrated state. Within one week of the aforementioned assessments, physical activity, diet patterns, sleep habits, and demographics were obtained with questionnaires, which were completed at home in coordination with both the primary caregiver and the participant. These questionnaires were administered using an online survey platform (Lime Survey, open source). In instances in which the computer-based survey was not feasible, a paper copy was given, which was later entered into the online Lime Survey by the investigator. This study format was implemented at each participating school. Only participants with full data sets were incorporated in the data and statistical analyses.

### 2.3. Primary Outcome: Cardiometabolic Disease Risk

Thirteen dependent variables were measured to reflect cardiometabolic health utilizing adiposity, pulse wave analysis (PWA), and cardiometabolic biochemical markers (Table 2). 

#### 2.3.1. Adiposity

Two adiposity-related variables were assessed: fat mass index (FMI) and waist-to-hip ratio (WHR). For FMI, fat mass (kg) was measured via multifrequency body impedance analysis (BodyStat Quadscan 4000, Isle of Man, UK). The use of BIA to determine adiposity is considered more reliable than other methods, such as skinfolds for children accordioning to a study conducted by Noradilah et al., 2016 [19]. The instrument was calibrated in accordance with the manufacturer’s instructions, and measurements were conducted according to standardized procedures. FMI was calculated by dividing the fat mass (kg) by height squared (m^2^).

#### 2.3.2. Pulse Wave Analysis

Peripheral systolic (SBP) and diastolic blood pressure (DBP), central systolic (aortic) blood pressure (CBP), heart rate (HR), and augmentation index (AIx) were measured utilizing the BP+ device (USCOM, Sydney, Australia) which has been validated in children and adolescents [20]. PWA was measured following 20 min of undisturbed rest, at which point oscillometric pressure waveforms were recorded by a single operator on the left upper arm following standard manufacturer guidelines [21]. Each measurement cycle lasted approximately 40 s, consisting of a brachial blood pressure recording and then a 10-s supra-systolic recording [21]. A corresponding aortic pressure waveform was generated using a validated transfer function, from which CBP was estimated [22]. The augmentation index was calculated from the supra-systolic waveform using the formula: AIx = [(P_2_ − P_0_)/(P_1_ − P_0_)] × 100%, where P_0_ denotes the pressure at the onset of the pulse, P_1_ the peak pressure of the incident wave, and P_2_ the peak pressure of the reflective wave. This index describes the relative height of the reflected pressure wave when compared to the incident waveform. Only recordings with a high signal quality were accepted (signal to noise > 3 dB). Two high-quality (high signal:noise ratio) measurements were taken within a 5-min period. A third reading was taken, and the mean taken of the closest two recordings if blood pressures or AIx’s were separated by >5 mmHg or 4%, respectively [21].

#### 2.3.3. Blood Biomarkers

Following PWA, cardiometabolic biochemical parameters were assessed using a traditional finger prick procedure. Capillary blood was extracted for assessment of fasting total cholesterol (TC), HDL-C, low-density lipoprotein (LDL-C), TG, serum glucose, and glycosylated hemoglobin (HbA1c). Biochemical measures were analyzed using portable glucose/lipid analyzers (CardioCheck PTS Diagnostics, IN, USA) and HbA1c (A1CNow+, PTS Diagnostics, Whitestown, IN, USA). 

### 2.4. Exposure Variables

#### 2.4.1. Cardiorespiratory Fitness

Cardiorespiratory fitness was estimated using the Maximal Multistage 20-Meter Shuttle Run Test (20-MST) and handgrip strength tests. The 20-MST is noninvasive and valid, without the need for a large space or specialized equipment or facilities. Further, according to a study by Wilkinson et al., 2010, the 20-MST is correlated with VO_2_ max (r = 0.91, *p* < 0.01) [23]. Additionally, this test is convenient for a school setting since many students can be tested simultaneously [24,25,26]. The 20-MST occurred at 10:00 a.m. on a Friday. Efforts were made in order to create testing environments in as similar conditions as possible based on the resources across the various schools. After warming up, stretching, and participating in a practice run, participants were instructed to run in groups of 10 between two lines that were set 20 m apart [27]. A speed of 8.5 km/h^−1^ was used as the starting pace, which increased by 0.5 km/h^−1^ for each successfully completed level. The speed was monitored by sounds emitted from a speaker [25]. Participants were cautioned the first time they did not get to the line in the allotted time with the audio signal and were then removed from the test if they did not reach the line for two successive runs or if the participant voluntarily ceased running [25]. Estimated VO_2_ max was calculated using Hamlin’s regression equation [25] which comprises variables including total distance covered, body fat percentage (body fat%), age, and maturity [28]:

[VO_2_ max (mL/kg) = 42.18 + (0.009 × beep test distance in meters [20 m]) + (−0.1762 × body fat%) + (−0.4091 × maturity)] (1)

The formula above has been validated previously on New Zealand children [25]. In accordance with the Cooper Institute FitnessGram^®^ cutoff points [29], a “healthy cardiorespiratory fitness zone” was reported if females achieved a VO_2_ max≥ 39 mL/kg/min and if males achieved ≥42 mL/kg/min. A VO_2_ max below those cutoff points was categorized as a “needs improvement fitness zone” for both sexes. 

#### 2.4.2. Muscular Fitness

Muscular fitness was estimated using the widely used handgrip test. A handgrip dynamometer (Camry, South El Monte, CA, USA) was used to assess each participant’s muscular strength. The method is rapid, noninvasive, simple to use, inexpensive, and of minimal risk, which are key factors when assessing a large group of children. The participants were seated with shoulders adducted and neutrally rotated, elbow flexed to 90 degrees, and their wrists in a neutral position (between 0- and 30-degrees extension and between 0 and 15 degrees ulnar deviation). Next, the participants placed their fingers around the dynamometer handle, the researcher counted down from three, and the participants were instructed to squeeze the dynamometer handle as hard as they could for three or more seconds. Each participant was given three attempts with each hand, alternating hands, and a one-minute recovery time between each attempt. Isometric handgrip strength was measured in kilograms, and the best score for each hand was recorded for analysis [30]. 

#### 2.4.3. Physical Activity and Sedentary Behavior

Physical activity and sedentary behavior were measured using the Youth Physical Activity Questionnaire (YPAQ), which is considered valid and reliable in children [31]. Participants and their caregiver(s) were asked to jointly complete the 47-item YPAQ to assess how many minutes per day participants were active and sedentary. The YPAQ measured the frequency, duration, and type of physical activities and sedentary behavior the participant took engaged in across the week leading up to data collection [32,33]. Activity type was recorded to classify activities as active movements or sedentary behavior. For example, playing football (soccer), walking for transportation, or bike riding were considered active, whereas television viewing, reading, and doing homework for school were considered sedentary. Frequency and duration were also recorded to estimate the total number of daily active and daily sedentary minutes, which enabled the calculation of the daily average and weekly total of active and sedentary minutes for each participant.

#### 2.4.4. Sleep

Three independent variables for sleep were assessed: social jetlag, sleep duration and sleep disturbances. Social jetlag was calculated as the absolute difference between the midpoints of sleep on week versus weekend days [34]. To determine average sleep duration, the participant’s caregiver(s) was/were asked to note what time their child usually went to bed and what time they usually got up on both school and weekend days. Single items of habitual school/weekday sleep show reasonable concurrent validity with actigraphy and diary data [35]. Average sleep duration was calculated using a ratio of 5 weekdays to 2 weekend days. Sleep disturbances were recorded using the 33-item Child Sleep Habits Questionnaire (CSHQ), which demonstrates adequate internal consistency, acceptable test–retest reliability, and discriminant validity [36]. The 33 questions were answered on a 7-point Likert—type scale from 0 (*never*) to 7 (*always*), with higher scores indicative of greater sleep disturbance. The CSHQ includes eight subscales that align with key sleep complaints relevant to this age group: bedtime resistance, sleep onset delay, sleep duration, sleep anxiety, night waking, parasomnias, sleep-disordered breathing, and daytime sleepiness. A Total Sleep Disturbances score was calculated as the sum of all CSHQ-scored questions, with a potential range of 33 to 99. A Total Sleep Disturbances score > 41 indicated a pediatric sleep disorder, as this cutoff point has been shown to accurately identify 80% of children with a clinically diagnosed sleep disorder. For this study, only the Total Sleep Disturbances score was analyzed [36].

#### 2.4.5. Nutrition

Food choice information was assessed using the Physical Activity, Exercise, Diet, and Lifestyle Study Food Frequency Questionnaire (PEDALS FFQ), comprised of 28 items. The PEDALS FFQ has been validated in this age group and shows acceptable reliability and validity [37]. In this study, these 28 items were aggregated into 21 groups, and principal component analysis (PCA), a statistical data reduction method, was conducted to identify components (patterns) from these 21 food groups. PCA restructures large data samples into new combined variables called principal components. The principal components account for variation in the sample, enabling the dietary data to be captured with fewer variables. Determining the number of components/patterns to be retained was based on eigenvalues > 1, identification of the “elbow” in the scree plot, and the interpretability of factors within components/patterns [38]. Three dietary components/patterns were identifiable: processed food, fruit and vegetables pattern, and breakfast foods patterns.

### 2.5. Covariates

Age (years/months), biological sex (female/male), and ethnicity (New Zealand European and Others, Māori, Pacific, not specified) were self-reported [39]. School decile was used as a measure of socioeconomic position. Briefly, schools with a greater proportion of students from low socioeconomic communities (decile 1) receive more funding than schools with fewer students from low socioeconomic communities (decile 10). 

### 2.6. Statistical Analysis

Statistical analyses were performed using R (Version 4.0.0). Raw data are presented as mean (standard deviation) and regression outcomes as standardized (β) betas (effect sizes). Using the β, the effect was adjudicated as trivial (<0.2), small (0.2–0.5), moderate (0.5–0.8), or large (>0.8). Additionally, point (two-sided *p*-value) and interval (95% confidence interval [CI]) estimates of statistical significance are presented, with two-sided *p*-values of <0.05. Factor analysis was used to obtain factor loadings (cardiometabolic factors) from the cardiometabolic variables. The principle of eigenvalues > 1 was used to determine the number of CMD risk factors (Table 2) that should be retained. More specifically, this principle, as determined by visual inspection of the scree plot, implies that if an eigenvalue is <1, the derived dimension captures less variance in the data than any sole variable. The factors then underwent orthogonal varimax rotation. Factor loadings (correlation between derived factors and underlying variables) were used for factor interpretation. A minimum loading of >|0.40| was employed since loadings below this threshold flag that a variable was not substantially contributing to a given factor. The cardiometabolic factors derived from factor analysis were blood pressure, cholesterol, adiposity, and metabolic. In order to create a cumulative CMD risk score, the factors were re-standardized and summed so that all measurements (e.g., all regression beta coefficients) were based on a 1 standard deviation unit change. Participants were then grouped into low-, normal-, and high-CMD risk, defined as having CMD risk scores of <−1, −1 to 1, and >1, respectively (Table 1 and Table 2). Statistical analyses were not performed to compare these CMD risk stratifications but simply to help characterize participants.

Linear mixed-effects models, with children nested within schools, were used to identify relationships among the exposure- (physical fitness and lifestyle factors) and outcome- (CMD risk score) variables [40]. For Model 1, a univariable analysis was conducted, in which each exposure variable was regressed against the CMD risk score. For each independent variable, linearity was explored by specifying the quadratic term. In the event of nonlinearity, to minimize collinearity, the independent variable was centered and then used to create the quadratic term. An independent variable was omitted from Model 2 if it did not significantly associate at alpha < 0.10 with the CMD risk score in Model 1. For Model 2, an unadjusted multivariable analysis was utilized in which all significant exposure variables were regressed against the CMD risk score. Model 3 was an adjusted version of Model 2, accounting for sex, ethnicity, age, and school decile. All regression models were assessed by examination of the model residuals plotted against their normal scores. The assumptions of normality and homoscedasticity were assessed via visual inspection of the frequency and residual distributions, respectively. To test for multicollinearity, variance inflation factors were compared to the recommended cut-point of 10.

Model 1 was a univariable analysis that used a single independent variable (CRF, handgrip strength, physical activity, sedentary minutes, sleep duration, social jetlag, sleep disturbance, processed foods, fruit and vegetable consumption, and breakfast food pattern) and one dependent variable (cardiometabolic factors: blood pressure, cholesterol, adiposity, and metabolic). Model 2 comprised all independent variables and one dependent variable (summed CMD risk score). Model 3 consisted of Model 2 with additional adjustments for age, ethnicity, sex, and school decile. Examination of residuals plotted against normal scores was conducted for all regression models.

## 3. Results

Out of the 392 participants who were included in the study, complete data was only present for 316, which were included in the analyses (Figure 1). Data sets that were incomplete were either missing questionnaires and/or CMD risk data. Categorical and continuous participant characteristics are presented in Table 1 (a) and (b).

### 3.1. Cardiometabolic Factor Correlations and Analysis

The factor analysis is summarized in Table 2. Using the minimum eigenvalue principle of >1, four dimensions (blood pressure, cholesterol, adiposity, and metabolic) were used in the factor analysis as described previously. Together, the four factors explained 56.8% of the variance in the measured variables. SBP, DBP, and CBP loaded positively onto the blood pressure factor. Total cholesterol, LDL-C, and AIx positively loaded onto the cholesterol factor. AIx, FMI, WHR, HR, and glucose positively loaded onto the adiposity factor. HDL-C (inversed to match directionality of other variables; i.e., higher = more unhealthy), FMI, triglycerides, and HbA1c loaded positively onto the carbohydrate-metabolic factor.

### 3.2. Univariate Models

**Figure 2:**
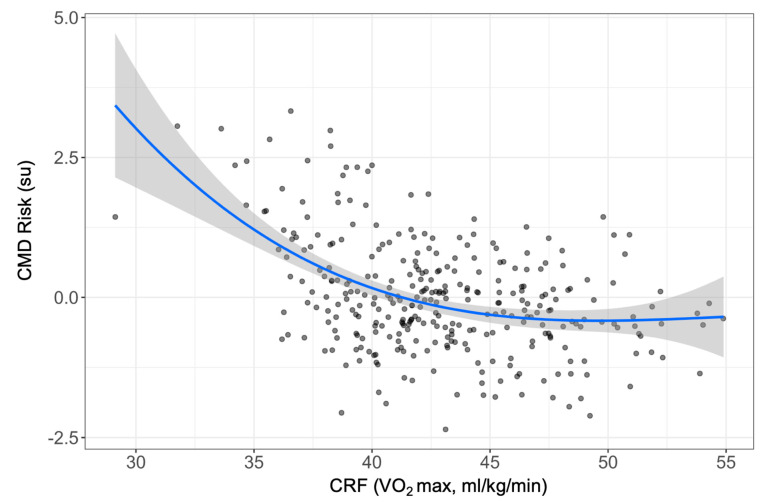
Association between Cardiorespiratory Fitness and Cardiometabolic Disease Risk. Scatterplot displaying the nonlinear relationship between CRF (VO_2_ max) and CMD risk. The *y*-axis (CMD risk score) is based on the re-standardized and summed results of the factor analysis of the CMD risk variables such that the CMD risk scores are based on a 1 standard deviation unit change. We considered CMD risk scores of <−1, −1 to 1, and >1 as low-, normal-, and high-CMD risk, respectively. Abbreviations: CMD, Cardiometabolic disease, CRF, cardiorespiratory fitness; su, standardized units, VO_2_ max, maximal volume of oxygen consumption (cardiorespiratory fitness).

Univariate outcomes (Model 1) are displayed in Table 3. Initially, each independent lifestyle factor was analyzed for association with each cardiometabolic factor derived from the factor analysis. The results showed that physical activity, sleep disturbances, average sleep duration, breakfast food pattern, and processed food was not strongly associated (*p* ≥ 0.10) with CMD risk factors. Based on our analysis plan, these factors were excluded from multivariable analysis since only independent factors that associated (*p* < 0.10) univariately with cardiometabolic factors were included. Additionally, the association of VO_2_ max with the CMD risk score was nonlinear (Figure 2). Therefore, VO_2_ max and the associated nonlinear/polynomial (VO_2_ max_Poly_) were used to account for nonlinearity. Based on visual inspection of Figure 2, it was observed that beyond 42 mL/kg/min, increases in VO_2_ max did not correspond with a change in CMD risk.

### 3.3. Multivariable Models

The unadjusted (Model 2) and adjusted (Model 3) multivariate analyses are shown in Table 3. In the unadjusted multivariable analyses (Model 2), the physical fitness variables, including VO_2_ max (β = −0.4, CI: −0.52, −0.32) and muscular fitness (β = −0.2, CI: 0.08, 0.28) were associated with the CMD risk score. Sedentary time was also associated with the CMD risk score (β = 0.1, CI: 0.03, 0.22). For the adjusted (sex, ethnicity, age, school decile) multivariable analyses (Model 3, Table 3), VO_2_ max was associated with the CMD risk score (β = −0.4, CI: −0.56, −0.34; Table 3). Sedentary time also remained associated with the CMD risk score (β = 0.1, *p* = 0.019, Table 3). 

## 4. Discussion

The purpose of this research was to study the relationships among CRF, muscular fitness, physical activity, sedentary behavior, nutrition, and sleep with CMD risk in preadolescents. After adjusting for possible confounding variables, CRF and sedentary behavior were associated with the CMD risk score, while physical activity, handgrip strength, processed food pattern, fruit and vegetable pattern, and social jetlag were not associated with CMD risk. After adjusting for sex, ethnicity, age, and school decile, CMD risk was associated with the CMD risk score (β = −0.4, CI: −0.56, −0.34; Table 3) and sedentary time (β = 0.1, *p* = 0.019, Table 3). Therefore, our findings suggest that enhancing CRF and decreasing sedentary behavior may be important CMD risk targets in preadolescent children.

### 4.1. Comparison with Other Studies

CRF was most strongly associated with CMD risk, followed by sedentary behavior. As will be discussed below, our belief is that these two factors represent different underlying biological constructs [41,42]. With respect to the former, CRF is a simple construct that represents multi-system cardiometabolic physiology and is a particularly useful target for health-based interventions in this population [43]. It should be recognized, however, that CRF was nonlinearly associated with CMD risk. Beyond ~42 mL/kg/min, an increase in VO_2_ max did not correspond with a change in CMD risk. This suggests that it may be particularly important to focus on improving CRF in children with a VO_2_ max below 42 mL/kg/min. Compared to normative data [44], a VO_2_ max of 42 mL/kg/min in children are classified as “fair” [45]. Therefore, “fair”, “poor”, or “very poor” VO_2_ max classification ^45^ is associated with higher CMD risk. These data are similar to other studies which have also demonstrated associations between lower VO_2_ max and CMD risk [46,47,48]. However, the present study provides continuous data to better elucidate the nonlinear association between VO_2_ max and CMD risk, which is lost when VO_2_ max is partitioned into classifications. 

Additionally, it is important to emphasize the importance of CRF relative to the narrative commonly touted in popular media, which is that obesity is the main lifestyle-based “culprit” associated with cardiometabolic disease risk in children [49]. At the same time, obesity is undoubtedly an important CMD risk factor that should be monitored and targeted. In support of this stance, our group previously showed that the relationship between fatness and CMD risk in preadolescents might be moderated by CRF, with fatness being associated with increased CMD risk in preadolescents with low, but not high CRF [3]. Collectively, children with low CRF are at greater risk of CMD and sustaining lower-than-average physical fitness levels in adulthood [50]. Additionally, it is important to note that VO_2_ max differs by biological sex, age (i.e., puberty stage), and associations of maturation, biological sex, and body fat with VO_2_ max have been previously demonstrated [51]. In the present study, despite the same weight (kg) between males and females, there were differences in body fat percentage between the sexes. At the onset of puberty, females tend to have higher body fat suggesting classifications for outcomes such as VO_2_ max should account for biological sex [52].

Like CRF, sedentary behavior was also associated with CMD risk. However, since sedentary behavior is associated with distinct CMD pathology compared to low CRF, we believe that sedentary behavior likely represents a separate latent construct and is a biologically distinct risk factor as compared to CRF (and physical [in]activity) [41,42]. Our findings build on prior research that suggests sedentary behavior may be a particularly important lifestyle factor to consider in the context of CMD risk. While preadolescent-specific data are sparse, adult sedentary behavior is an evolving health risk behavior for the development of chronic diseases, including major CMDs. Recent independent studies suggest that sedentary behavior is strongly associated with diabetes, obesity, cardiovascular disease (CVD), and mortality [53,54,55,56,57,58,59]. Among children and adolescents, (a) higher duration of television viewing has been associated with higher blood pressure [60,61]; (b) combined sedentary time and video game playing has been associated with lower CRF [62]; and (c) higher screen time duration associated with decreased muscular strength [63]. In sum, SB is a biologically novel CMD risk factor that is likely an important contributor to CMD risk in preadolescents. We direct readers interested in practical suggestions on how to target SB in preadolescents to our prior commentary on this topic [64].

### 4.2. Limitations

Several limitations should be mentioned to provide further context to the current findings. As this was a cross-sectional study, causality cannot be determined. Future longitudinal research is needed to better understand the directionality of associations among physical fitness, physical activity, sedentary behavior, nutrition, and sleep with CMD risk in preadolescents. This cross-sectional study is a necessary preliminary starting point before devoting time and resources to expensive longitudinal studies. Also, since data collection occurred in group settings at primary schools, factors such as audible noise, facility limitations (e.g., limited space and privacy), distractions, interruptions, and the weather could not be controlled or measured. These factors could have influenced data collection on blood pressure, HR, as well as the focus and attention span of the participants [65]. Finally, this study’s sample was limited to preadolescents in New Zealand, which is likely not generalizable to preadolescents in other geographical regions. For example, dietary intake (Mediterranean diet vs. Western diet), types of physical activities (soccer vs. American football), and social-ecological factors (e.g., neighborhood safety and access to affordable healthcare) may affect preadolescent physiology uniquely and vary by geographical region. Thus, further research is needed to determine whether these findings would be generalizable to preadolescents globally. Despite these limitations, noteworthy strengths of this study were that the participants reflected a large, diverse (including both in terms of race/ethnicity and socioeconomic position) sample of preadolescent children from various New Zealand regions and that a wide-ranging and concurrent measurement of multiple important lifestyle factors and CMD risk biomarkers was undertaken.

## 5. Conclusions

CRF and sedentary behavior are particularly important targets for mitigating CMD risk in preadolescent children. Importantly, CRF and sedentary behavior reflect distinct underlying biological constructs in terms of how they impact CMD risk and should be targeted as such. We do not believe that CRF and sedentary behavior are the *only* important lifestyle factors in the context of CMD risk in preadolescence—but rather that these factors should be prioritized as specific, focal targets for public health interventions in this population. Future interventions should seek to enhance CRF and reduce sedentary behavior in preadolescent children to offset the risk and associated societal and economic burden of CMDs.

## Figures and Tables

**Table 1:** (a) Overall and Risk Stratified Participant Characteristics for Categorical Variables. (b) Overall and Risk Stratified Participant Characteristics for Continuous Variables.

(a)								
			Stratified by CMD Risk Score					
	Total		Low		Normal		High	
	*n*	%	*n*	%	*n*	%	*n*	%
Categorical Variables								
Ethnicity								
European	257	82	35	14	191	74	31	12
Māori-Pacific Islander	56	18	6	11	34	61	16	29
School Year								
4	69	22	13	18	52	75	4	6
5	88	28	9	10	71	81	8	9
6	96	30	15	16	60	63	21	22
7	63	20	4	6	44	70	15	24
Decile								
Low (≤5)	162	51	23	14	109	67	30	19
High (>5)	154	49	18	12	118	77	18	12
Weight Status								
Overweight	89	28	1	1	59	66	29	33
Non-Overweight	227	72	40	18	168	74	19	8
Fitness level								
VO_2_ max (mL/kg/min)	42.9	4.4	45.1	4	43.3	4	39.4	5
VO_2_ max Low	232	73	38	16	178	77	16	7
VO_2_ max High	84	27	3	4	49	58	32	38
**(b)**								
			**Stratified by CMD Risk Score **					
	**Total**		**Low**		**Normal**		**Low**	
	**Mean**	**SD**	**Mean**	** SD**	**Mean**	**SD**	**Mean**	** SD**
Continuous Variables								
Age (years)	9.6	1.1	9.2	1.1	9.5	1.2	10.0	0.9
Body Fatness								
Weight (kg)	34.4	9.2	30.7	5.6	33.1	7.3	43.9	13.0
Body Fat (%)	19.7	9.4	13.1	5.3	18.7	7.5	29.9	12.2
Fat Mass Index (fat mass/m^2^)	3.65	2.4	2.1	0.9	3.3	1.6	6.7	3.7
Waist-to-Hip Ratio	0.8	0.1	0.8	0.1	0.8	0.0	0.9	0.1
Physical Activity & Sedentary Behavior								
Physical Activity (min)	166.0	137.0	190.2	151.3	162.8	131.4	162.2	151.9
Sedentary Behavior (min)	282.0	208.0	230.5	158.1	286.4	214.1	308.3	212.5
Sleep								
Average Sleep Duration (h)	10.1	0.8	10.4	0.7	10.1	0.8	10.1	0.9
Social Jetlag (h)	0.7	0.5	0.6	0.4	0.7	0.5	0.9	0.6
Sleep Disturbances	40.2	5.9	39.5	5.5	40.2	6.0	41.0	6.0
Dietary Habits								
Processed Food	0.0	1.9	0.4	2.3	−0.1	1.4	0.5	3.1
Fruit and Vegetable Pattern	0.1	1.6	0.5	1.8	0.1	1.5	−0.5	1.5
Breakfast Food	0.0	1.3	0.2	1.3	0.0	1.3	−0.1	1.2
Cardiometabolic Risk								
Systolic Blood Pressure (mmHg)	100.8	7.7	94.7	5.0	100.4	6.9	108.4	7.5
Diastolic Blood Pressure (mmHg)	61.6	6.2	55.8	5.0	61.6	5.4	66.9	6.0
Central Blood Pressure (mmHg)	93.3	7.7	86.9	6.3	93.1	6.9	99.9	7.5
Augmentation Index (%)	55.8	15.3	57.1	13.6	56.5	15.3	51.3	16.1
Heart Rate (bpm)	74.8	11.7	67.9	10.4	75.1	11.2	79.3	12.8
Fasting Blood Glucose (mmol/L)	5.0	0.4	5.0	0.4	5.0	0.4	5.1	0.4
Glycosylated Hemoglobin (%)	5.1	0.3	5.0	0.3	5.1	0.3	5.3	0.4
Total Cholesterol (mmol/L)	3.6	0.6	3.1	0.4	3.6	0.5	4.0	0.8
HDL Cholesterol (mmol/L)	1.5	0.4	1.4	0.3	1.5	0.4	1.5	0.4
LDL Cholesterol (mmol/L)	1.9	0.5	1.6	0.4	1.8	0.5	2.2	0.6
Triglycerides (mmol/L)	0.9	0.4	0.8	0.2	0.8	0.4	1.1	0.6

Participant Characteristics for Categorical and Continuous variables. The table presents overall participant characteristics as well as participant characteristics stratified by cardiometabolic disease risk status (low, normal, or high). Abbreviations: CMD; Cardiometabolic disease; HDL, High-density lipoprotein cholesterol; LDL, Low-density lipoprotein cholesterol; VO_2_ max, the maximal volume of oxygen consumption (cardiorespiratory fitness); VO_2_ max_Poly,_ maximal oxygen consumption (cardiorespiratory fitness) with the polynomial term.

**Table 2:** Results of Factor Analysis to Identify Cardiometabolic Patterns.

	Factor 1	Factor 2	Factor 3	Factor 4	Comm.
	BP	CHO	Adiposity	Carb-Met	
SBP	**0.92**	0.04	0.16	−0.03	0.128
DBP	**0.88**	−0.01	0.05	0.03	0.218
cSBP	**0.94**	0.04	−0.01	0.02	0.122
CHO	0.04	**0.92**	0.00	0.00	0.148
LDL-C	0.00	**0.73**	−0.02	0.07	0.460
HDL-C	−0.04	**−0.50**	0.03	**0.50**	0.493
AIx	0.25	−0.03	**−0.68**	0.13	0.458
FMI	0.25	−0.09	**0.56**	**0.46**	0.410
WHR	0.06	0.03	**0.50**	0.29	0.663
HR	0.27	0.07	**0.48**	−0.19	0.652
Glucose	0.07	−0.10	**0.48**	−0.14	0.739
Triglycerides	0.14	−0.05	−0.13	**0.65**	0.536
HbA1c	−0.17	0.12	−0.05	**0.61**	0.587
Eigenvalue	2.8	1.7	1.5	1.4	
% Variance Explained	21.2	12.9	11.8	10.9	
Cumulative Variance	21.2	34.1	45.9	56.8	
KMO					0.56
Bartlett’s Test					<0.001
Bold numbers represent variables with a factor loading > |0.4|.					
components retained based on an eigenvalue of 1					

This table displays the results of the factor analysis of the 13 cardiometabolic risk variables (adiposity, pulse wave analysis, and biochemical markers), which determined the cardiometabolic disease risk score. Abbreviations: AIx, Augmentation index; BP, Blood pressure; cSBP, Carb-Met; Carbohydrate-metabolic; Central systolic blood pressure; CHO, Cholesterol; Comm, Communality (uniqueness); DBP, Diastolic blood pressure; FMI, Fat mass index; HbA1c, glycosylated hemoglobin; HDL, High-density lipoprotein cholesterol; HR, Heart rate; KMO, Kaiser-Meyer-Olkin Test, LDL, Low-density lipoprotein cholesterol; SBP, systolic blood pressure; WHR, Waist-to-hip ratio.

**Table 3:** Associations between lifestyle factors and CMD risk.

	β	LCI	UCI	*p*-Value
Univariable				
VO_2_ max	−0.44	−0.54	−0.34	**< 0.001**
VO_2_ max_Poly_	0.17	0.10	0.25	**<0.001**
Strength	0.15	0.04	0.26	**0.007**
Physical Activity	0.00	−0.11	0.11	0.974
Sedentary	0.15	0.04	0.26	**0.006**
Sleep Duration	−0.11	−0.22	0.00	0.052
Social Jetlag	0.13	0.02	0.24	**0.019**
Sleep Disturbance	0.09	−0.02	0.20	0.112
Processed Foods	0.04	−0.08	0.15	0.528
Fruit/Veg	−0.17	−0.28	−0.06	**0.003**
Breakfast	−0.07	−0.18	0.04	0.239
**Multivariable Model 1**				
VO_2_ max	−0.42	−0.52	−0.32	**<0.001**
VO_2_ max_Poly_	0.15	0.08	0.22	**<0.001**
Strength	0.18	0.08	0.28	**<0.001**
Sedentary	0.12	0.03	0.22	**0.013**
Sleep Duration	−0.05	−0.15	0.05	0.328
Social Jetlag	−0.01	−0.11	0.09	0.832
Fruit/Veg	−0.02	−0.12	0.08	0.717
**Multivariable Model 2**				
VO_2_ max	−0.45	−0.56	−0.34	**<0.001**
VO_2_ max_Poly_	0.19	0.11	0.27	**<0.001**
Strength	0.09	−0.02	0.21	0.113
Sedentary	0.12	0.02	0.21	**0.019**
Sleep Duration	−0.01	−0.11	0.08	0.766
Social Jetlag	−0.02	−0.12	0.08	0.662
Fruit/Veg	−0.03	−0.13	0.07	0.520

This displays associations between lifestyle factors and CMD risk. Associations were derived from the univariate, unadjusted multivariate, and adjusted multivariate regression analyses. Abbreviations: Fruit/veg, fruit and vegetable pattern; LCI, Lower 95% confidence interval; UCI, Upper 95% confidence interval, VO_2_ max, maximal volume of oxygen consumption (cardiorespiratory fitness), VO_2_ max_Poly,_ maximal oxygen consumption (cardiorespiratory fitness) with the polynomial term.

## Data Availability

Reduced and analyzed data are reported in the manuscript. The full raw data from this study are available on request from the corresponding author.
